# Study on improvement of the performance parameters of a novel 0.41–0.47 THz on-chip antenna based on metasurface concept realized on 50 μm GaAs-layer

**DOI:** 10.1038/s41598-020-68105-z

**Published:** 2020-07-03

**Authors:** Mohammad Alibakhshikenari, Bal S. Virdee, Chan H. See, Pancham Shukla, Shahram Salekzamankhani, Raed A. Abd-Alhameed, Francisco Falcone, Ernesto Limiti

**Affiliations:** 1 Electronic Engineering Department, University of Rome “Tor Vergata”, Via del Politecnico 1, 00133 Rome, Italy; 2 Center for Communications Technology, School of Computing and Digital Media, London Metropolitan University, London, N7 8DB UK; 3 School of Engineering and The Built Environment, Edinburgh Napier University, Merchiston Campus, 10 Colinton Road, Edinburgh, EH10 5DT UK; 4 School of Engineering, University of Bolton, Deane Road, Bolton, BL3 5AB UK; 5 Faculty of Engineering and Informatics, University of Bradford, Bradford, BD7 1DP West Yorkshire UK; 6 Electric and Electronic Engineering Department, Universidad Pública de Navarra, Pamplona, Spain

**Keywords:** Engineering, Electrical and electronic engineering

## Abstract

A feasibility study is presented on the performance parameters of a novel on-chip antenna based on metasurface technology at terahertz band. The proposed metasurface on-chip antenna is constructed on an electrically thin high-permittivity gallium arsenide (GaAs) substrate layer. Metasurface is implemented by engraving slot-lines on an array of 11 × 11 circular patches fabricated on the top layer of the GaAs substrate and metallic via-holes implemented in the central patch of each row constituting the array, which connects the patch to the leaky-wave open-ended feeding slot-lines running underneath the patches. The slot-lines are connected to each other with a slit. A waveguide port is used to excite the array via slot-lines that couple the electromagnetic energy to the patches. The metasurface on-chip antenna is shown to exhibit an average measured gain in excess of 10 dBi and radiation efficiency above 60% over a wide frequency range from 0.41 to 0.47 THz, which is significant development over other on-chip antenna techniques reported to date. Dimensions of the antenna are 8.6 × 8.6 × 0.0503 mm^3^. The results show that the proposed GaAs-based metasurface on-chip antenna is viable for applications in terahertz integrated circuits.

## Introduction

The terahertz (THz) frequency band that spans the frequencies between 0.1 and 10 THz offers potential applications in various disciplines including medical science^1^, imaging^2^, defence and security^3^, time-domain spectroscopy^4^, astronomy^5^, agriculture^6^, and wireless communication systems^7^. Antennas based on Planar Fabry–Perot cavity have been demonstrated at THz band and such antennas have a highly directive radiation characteristic^8,9^. Unfortunately, the design and fabrication these antennas can be complex, and their radiation efficiency is relatively low especially when implemented on high permittivity substrates^10^. Nevertheless, the authors in^11^ have successfully demonstrated a Fabry–Perot cavity antenna at THz band that is relatively easier to fabricate and exhibits high directivity and efficiency performance. At terahertz frequency band an electrically thick substrate establishes unwanted resonance in the substrate. It has been shown that these resonances can be avoided by simply decreasing the thickness of the substrate by λ_0_/20, where λ_0_ represents the wavelength of the free-space^12^.

Metasurface can essentially be created by distributing electrically small scattering artefacts over the surface of a dielectric medium that essentially perturb the propagation of electromagnetic waves^13^. In fact, the geometrical shape of the scattering artefacts determines the electromagnetic properties of the metamaterial^14^. Antennas implemented using metamaterial or metasurface structures have been shown to improve the performance of the antenna in terms of radiation gain, radiation efficiency, radiation pattern, and bandwidth^15^. Results of these investigations reveals that this technology can be applied to realize terahertz antennas making them viable for practical applications.

THz signals experience much greater attenuation and atmospheric loss in comparison with the conventional microwave links. Hence, antenna structures with high gain and high efficiency specifications are essential in the THz region. This paper presents a feasibility study of a THz on-chip antenna based on metasurface concept to improve its bandwidth, radiation gain and efficiency characteristics. The proposed metasurface on-chip antenna operates at a much higher frequency (410–470 GHz), which to the authors’ knowledge is demonstrated for the first time.

## Design process of the metasurface on-chip antenna

**Fig. 1:**
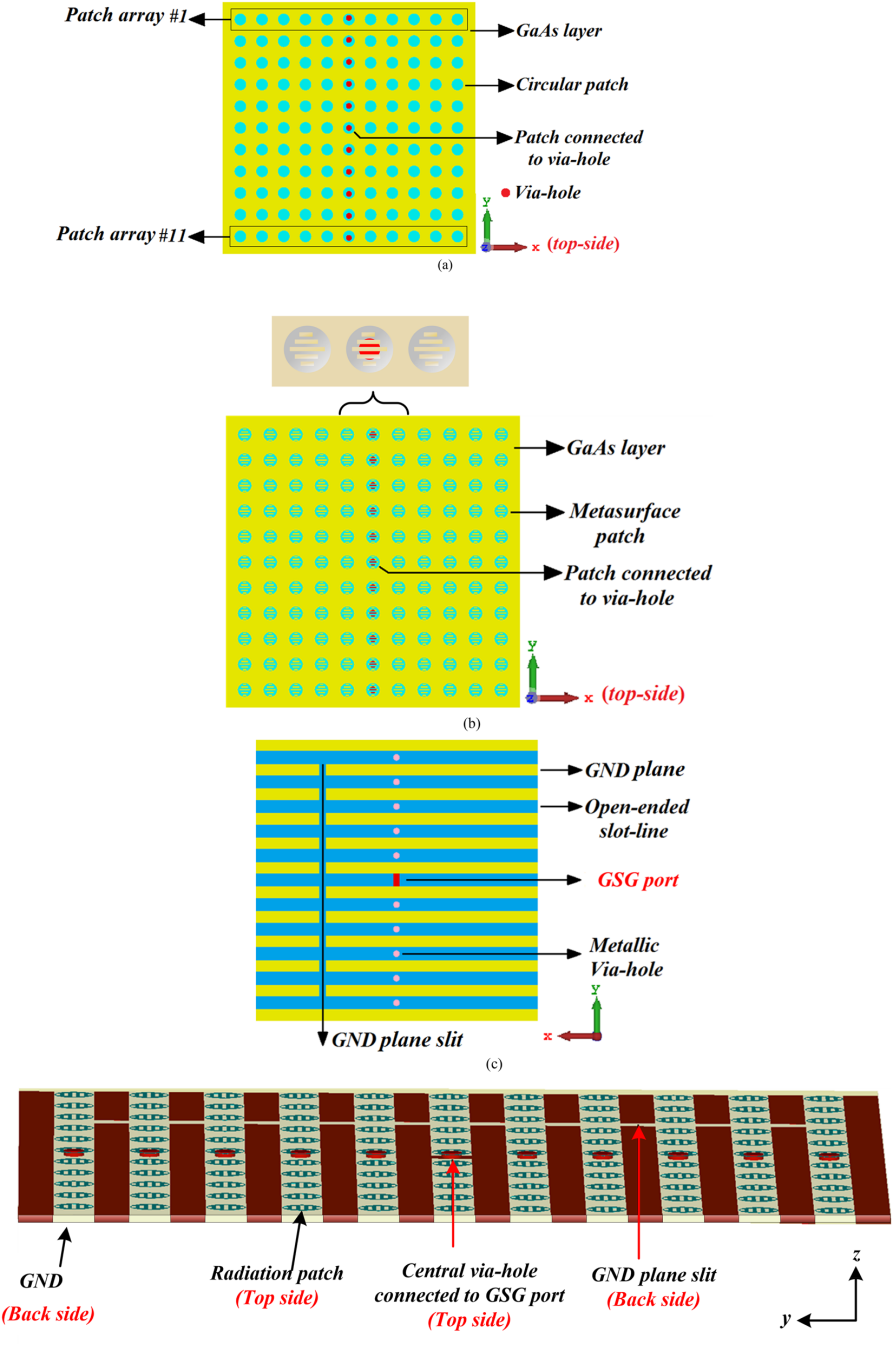

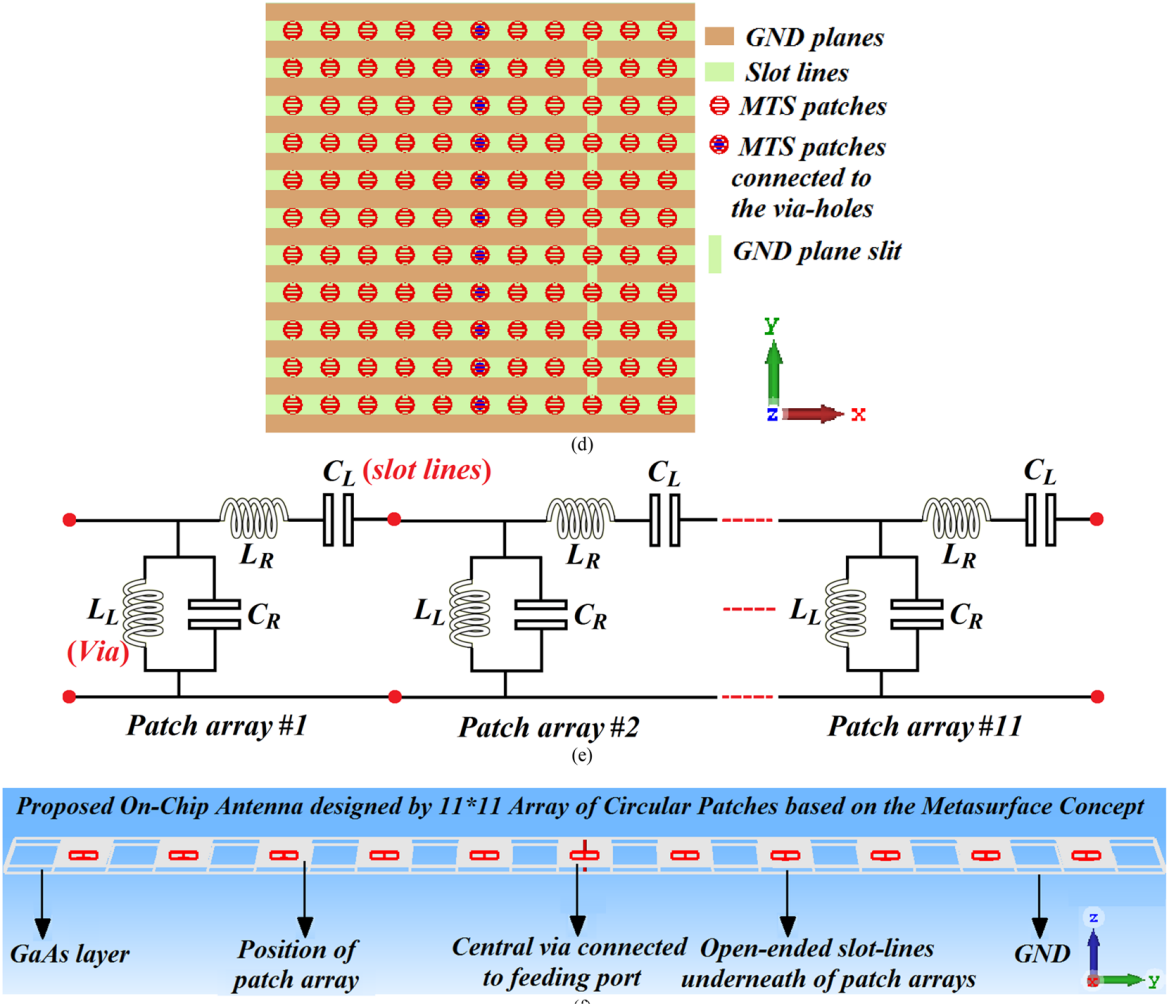
(**a**) Layout of the reference on-chip antenna containing an array of 11 × 11 circular patches without metasurface, top-view, (**b**) layout of the proposed on-chip antenna designed by an array of 11 × 11 circular patches with metasurface, top-view, (**c**) back-side of the both reference and proposed on-chip antenna structures. The GSG port is connected to the central via-hole and all metallic via-holes are electromagnetically connected to each other by the GND plane slit. (**d**) 3D isometric-view of the proposed on-chip antenna structure based on the metasurface concept, (MTS represents the metasurface), (**e**) equivalent circuit model of the proposed on-chip antenna, and (**f**) schematic view of the proposed structure.

Configuration of the reference on-chip antenna comprising an array of 11 × 11 circular patches without the metasurface slots is shown in Fig. 1a. The radiation elements of the reference on-chip antenna consist of circular patches implemented on an electrically thin, high-permittivity gallium arsenide (GaAs) layer. Similarly, the configuration of the proposed on-chip antenna in Fig. 1b consists of an array of 11 × 11 circular patches however these patches are embedded with slot-lines of various lengths to create a metasurface structure. In both cases the antennas are constructed by stacking together layers of metallization-GaAs-metallization. Each central radiation patch is punctured with a metallic via-hole at its center thus creating an RF path that connects the patch with the open-ended slot-line in the ground-plane through the GaAs substrate layer. Consequently, the antenna structure is excited through the open-ended narrow slot-lines that are patterned on the bottom-side of the GaAs substrate layer, which are aligned exactly under each row of the radiation patch arrays constituting the antenna, as shown in Fig. 1c,d. In the proposed feeding mechanism, the central metallic via-hole is connected to a coplanar waveguide (CPW) port and then all metallic via-holes are electromagnetically connected to each other through the ground-plane slit as shown in Fig. 1c. When the CPW port is excited it causes the electromagnetic energy to flow over the leaky-wave open-ended slot-lines and this energy is coupled to the circular patch arrays through the metallic via-holes causing the metasurface antenna to emit radiation. It will be evident in the Section III that the 2D metasurface structure essentially increases the effective aperture area of the antenna that enhances its radiation characteristics without increasing the antenna’s physical dimensions. Also, the surface-waves and the substrate losses are significantly suppressed by utilizing the proposed electromagnetic coupling feed mechanism to stimulate the radiation patches, which results in improvement of the antenna's performance in terms of radiation gain and efficiency over the operating frequency band.

The GaAs substrate employed has a dielectric constant of ${\varepsilon }_{r}$=12.9, loss-tangent of tan $\delta$=0.006, and thickness of *h* = 50 $\mu \text{m}$ (~ λ_0_/13, where λ_0_ is the wavelength of the free-space centered at 0.44 THz). Conductive elements in the on-chip antenna structures are Aluminium that had a thickness of 0.35 $\mu \text{m}$ and a conductivity of 3.56 × 10^7^ S/m. Both reference on-chip antenna and the proposed on-chip antenna structures have identical dimensions of 8.6 × 8.6 × 0.0503 mm^3^.

In the proposed 2D metasurface structure the slot-lines essentially behave as series left-handed capacitance resulting from the slot layer^16^. The proposed metasurface includes a metallic via-hole in the central patch of each row of patches that connects the top layer (radiation patches) to the bottom layer (ground-plane) through GaAs substrate layer. This introduces a shunt left-handed inductance. The structure also introduces unwanted parasitic effects resulting in the form of shunt right-handed capacitance and series right-handed inductance^17^. The shunt right-handed capacitance is due to the gap capacitance created between the radiation patches and the ground-plane, and the series right-handed inductance is created by the unavoidable surface currents^18^. The equivalent circuit model of the proposed on-chip antenna applying the metasurface principle is presented in Fig. 1e. Additionally, its schematic view is exhibited in Fig. 1f to better recognizing its constructional elements.

The antenna structure was modelled on a commercially available 3D EM full-wave solver (CST Microwave Studio™) using finite integration technique in the time domain. The antenna structure was optimized for a wide impedance bandwidth, radiation gain and efficiency performance. To better understanding the metasurface effects on the performance of the on-chip antenna, a reference on-chip antenna with no metasurface consisting of just the circular patches with no slot-lines, shown in Fig. 1a, was first analysed and compared with an on-chip antenna with metasurface structure. In section III, it is shown that with the metasurface there is an average improvement of 10.8% and 39.2% in the gain and radiation efficiency, respectively.

The simulation analyses conducted revealed that the gap between the outermost circular patches and the edge of the substrate is important. The gap should be approximately equal to the space between two adjacent patches to prevent destructive interference in the lateral plane resulting from diffracted waves from the edge of the substrate.

Antenna gain as a function of frequency was investigated for the various antenna matrix sizes. As expected, the gain of the antenna is a function of the matrix size of the radiating elements however the gain plateaus with increasing matrix size. This can be explained with a leaky-wave interpretation of this structure, i.e. once the propagating leaky (complex) wave-number is found. The attenuation constant of the leaky mode will determine the minimum antenna size *L* to radiate a given radiation efficiency, i.e. $\eta =1-{e}^{-\alpha L}$^19^.

Dimensions of the optimized on-chip antenna constructed of an array of 11 × 11 circular patches are thus: gap between patches is 200 μm, patch radius is 200 μm, via-hole radius is 100 μm, slot width is 40 μm, length and width of the open-ended slot-lines are 8.6 mm and 0.4 mm, respectively.

## Metasurface on-chip antenna performance

**Fig.2:**
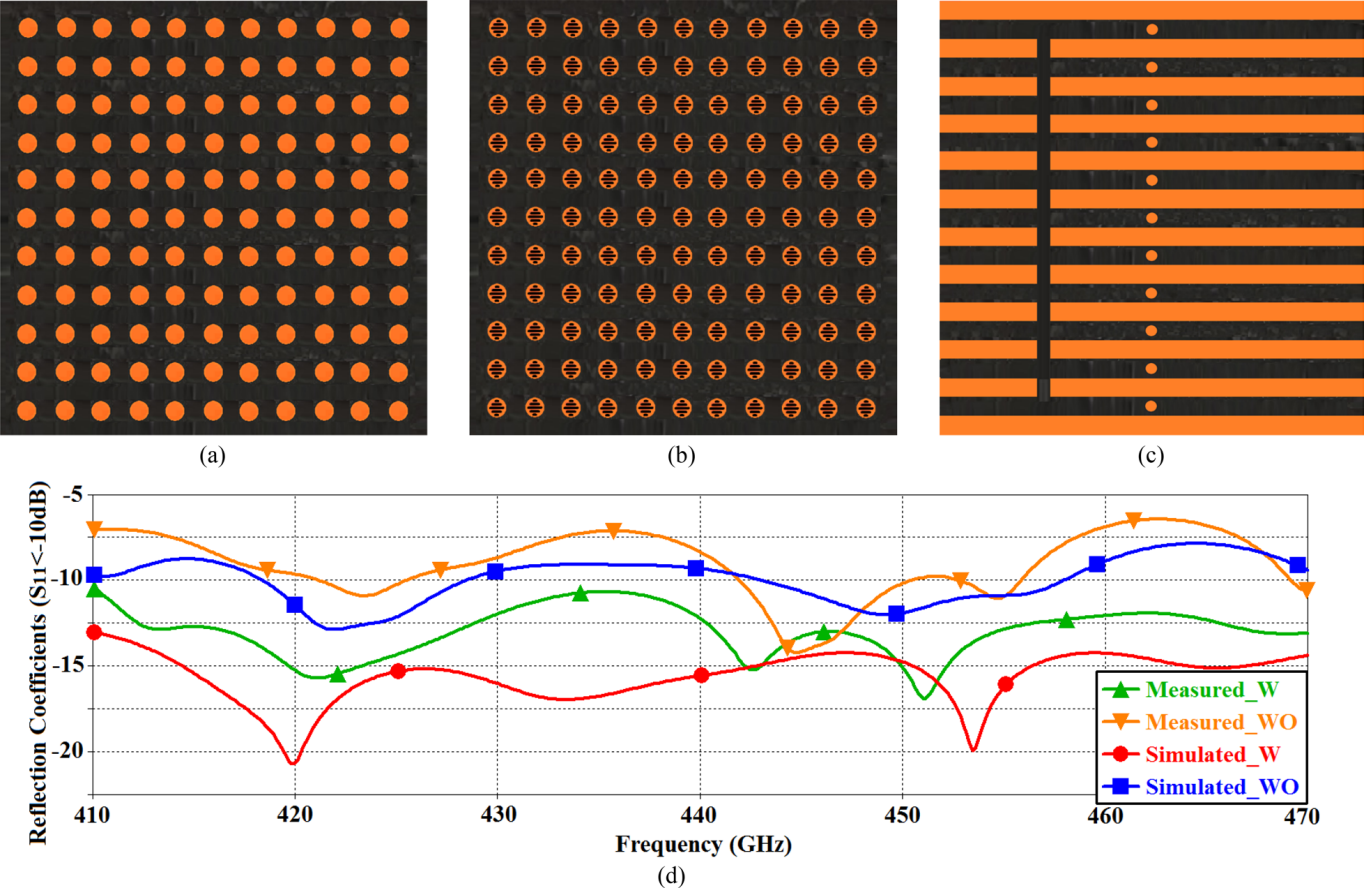
Fabricated prototypes of the reference, i.e. without (WO) metasurface slots, and the proposed, i.e. with (W) metasurface slots, on-chip antennas. (**a**) Top-view of the reference on-chip antenna, (**b**) top-view of the proposed on-chip antenna, (**c**) back-view of both op-chip antennas, and (**d**) the simulated and measured reflection-coefficient response.

Fabricated prototypes of the reference and the proposed on-chip antennas implemented through an array of 11 × 11 circular patches and their impedance bandwidth performance are shown in Fig. 2. The characteristics of the antenna were measured using a compact antenna test range as described in^25^. From the reflection-coefficient curves it is evident that after applying the metasurface the antenna's impedance bandwidth and impedance matching performance significantly improve. The proposed on-chip antenna with metasurface slot-lines has a measured impedance bandwidth from 0.41 to 0.47 THz for S_11_ < − 10 dB, which corresponds to a fractional bandwidth of 13.63%. The discrepancy observed between the measured and simulated results is due to (1) the unknown dielectric loss-tangent over the required frequency range in the foundry’s design kit when the 3D model of the antenna was constructed; (2) manufacturing tolerances; and (3) feed mismatch losses.

**Fig.3:**
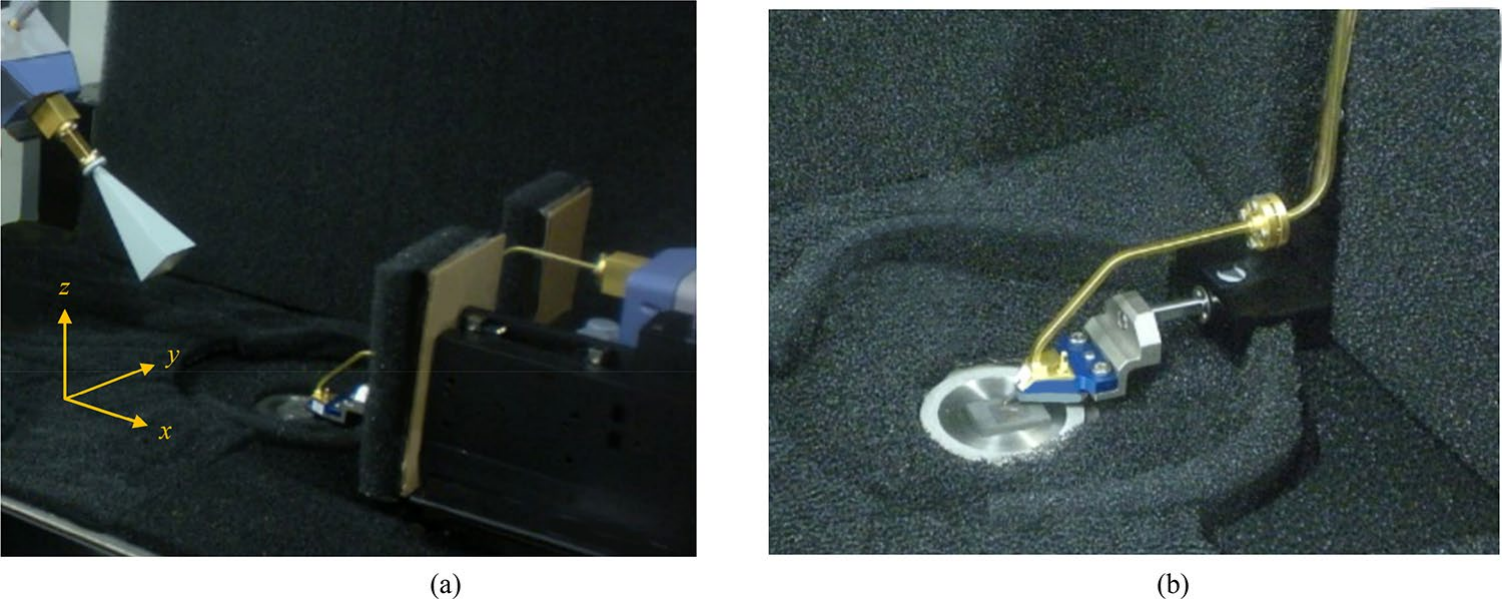
(**a**) The terahertz antenna measurement system with the attached horn antenna on the receiver; and (**b**) RF absorber material seen as black spongy sheets were added to all surfaces in the antenna measurement system to reduce multipath reflections. The on-chip antenna was placed on a Cascade Microtech rigid microwave absorber and excited using the ground-signal-ground (GSG) radio frequency (RF) probe.

The radiation characteristics of the on-chip antenna was measured using a compact antenna test range as explained in^20^. The IEEE Standard Test Procedures for Antennas^21^ was used to construct an accurate far-field antenna measurement system in a probe station environment. The antenna prototype was placed in a fixed position and made to transmit a constant power level. A receiver antenna with known gain was then used to measure the received power, polarization and power gain. The source antenna was positioned at various pointing angles with respect to the on-chip antenna and maintaining a constant distance. This configuration was used in the probe station antenna measurement system since the proposed prototype antenna is an on-chip antenna and must remain in a fixed position. The antenna measurement system with the attached horn antenna on the receiver is shown in Fig. 3a. To reduce multipath reflections in the test environment, radio frequency (RF) absorbing material was applied to nearly all metallic surfaces and objects on the probe station as shown in Fig. 3b. A vacuum pump was used to hold down the chip to the rigid microwave absorber while the RF probe touched down.

**Fig.4:**
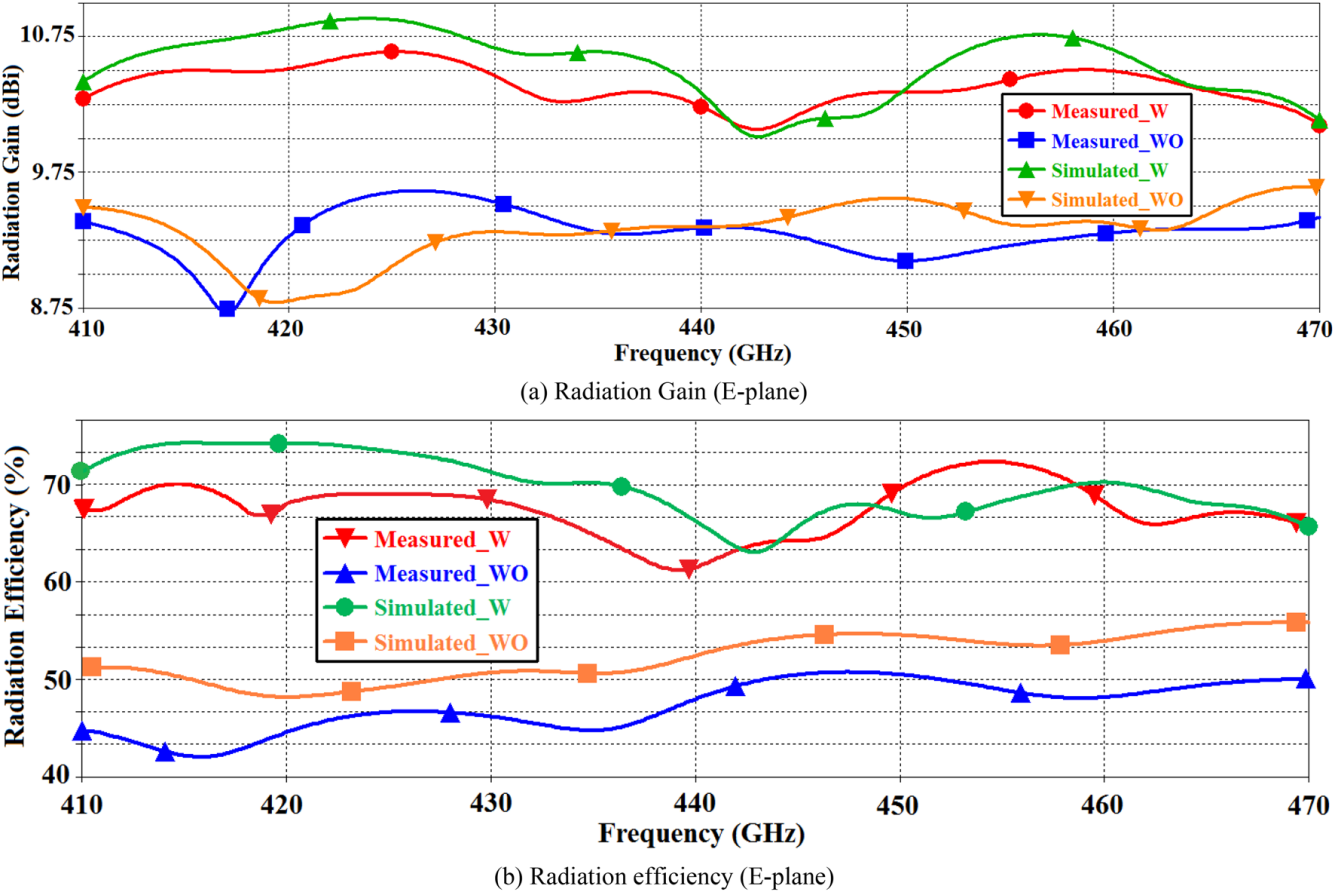
The simulated and measured radiation gain and efficiency plots without (WO) and with (W) metasurface slot-lines.

The simulated and measured radiation gain and efficiency performance of the reference on-chip antenna (without metasurface) and the proposed on-chip antenna (with metasurface) are shown in Fig. 4. These responses show that application of metasurface results in significant improvement in the gain and radiation efficiency over a wide frequency range from 0.41 to 0.47 THz. The average gain measured over this frequency range is 10 dBi with an optimum value of 10.64 dBi at 0.425 THz. Correspondingly, the proposed on-chip antenna exhibits efficiency of above 60% with an optimum value of 72.5% at 0.455 THz.

**Fig.5:**
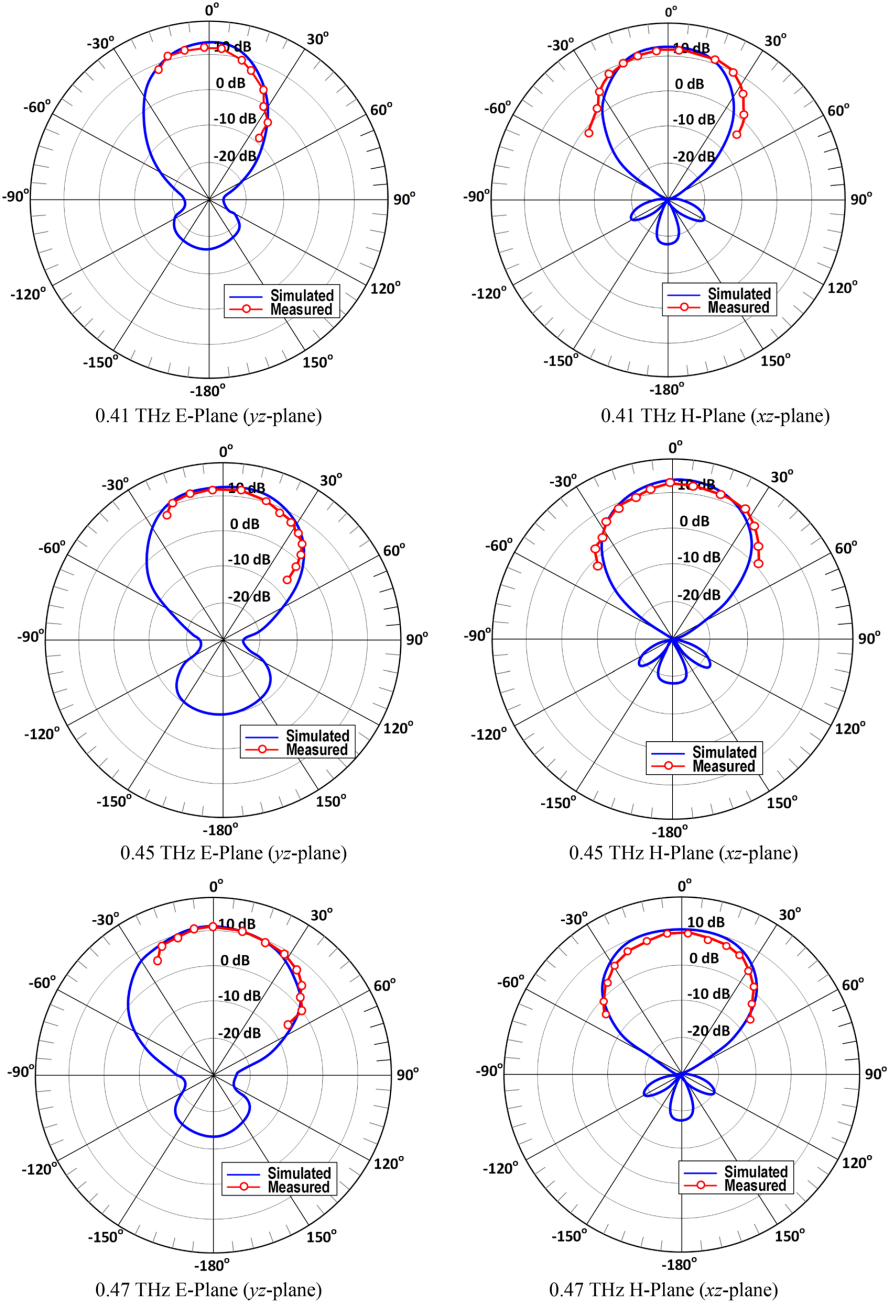
Simulated and measured E- and H-planes radiation patterns of the proposed on-chip antenna modelled by an array of 11 × 11 circular patches at 0.41 THz, 0.45 THz and 0.47 THz.

The simulated and measured radiation patterns of the metasurface on-chip antenna at 0.41, 0.45, and 0.47 THz are shown in Fig. 5. The measured results show the antenna generates a directional radiation pattern in both E-plane and H-plane and exhibits a wide 3 dB beamwidth over its operating frequency range. This is due to strong current flows across the open-ended slot-lines producing strong fields that result in a wider beamwidth. The simulation results show the antenna’s back lobes are relatively small compared to the main beam resulting in a high front-to-back ratio. Since the radiation pattern of the conventional slot is bi-directional, a back-reflector is often applied to prevent the back radiation from interfering with other systems.

Performance parameters of the proposed GaAs-based on-chip antenna with metasurface slot-lines is compared with other recently published millimeter-wave antennas in Table 1. The comparison shows that the proposed metasurface on-chip antenna operates at a much higher frequency from 410 to 470 GHz, which to the authors’ knowledge is demonstrated for the first time. In addition, the proposed antenna has comparable gain and radiation efficiency to the references cited in Table 1. It is worth to comment that, in order to provide deep and clear insight in relation with the metasurface effects on the performance parameters, the proposed on-chip antenna has been constructed of an array of 11 × 11 circular patches, which made its length and width larger than the cited works in Table 1. However, the proposed on-chip antenna is less complex and cost effective to implement in practice, which makes it a viable candidate for applications in terahertz integrated circuits.

**Table 1:** Salient features of the proposed metasurface on-chip antenna compared with recent publications.

References	Type	BW/[Freq. range] (GHz)	Gain (dBi)	Eff. (%)	Size	Process
^22^	Bowtie-slot	15/[90–105]	Max. −1.78	–	0.71 × 0.31 × 0.65 mm^3^	IHP 0.13-μm Bi-CMOS
^23^	Differential-fed	20/[50–70]	Max. − 3.2	–	1.5 × 1.5 × 0.3 mm^3^	0.18-μm
^24^	Ring-shaped monopole	20/[50–70]	Max. 0.02	Max. 35	–	CMOS 0.18-μm
^25^	Circular open-loop	10/57–67]	Max. − 4.4	–	1.8 × 1.8 × 0.3 mm^3^	CMOS 0.18-μm
^26^	AMC embedded squared slot antenna	51/[15–66]	Max. 2	–	1.44 × 1.1 mm^2^	CMOS 0.09-μm
^27^	Monopole	25/[45–70]	Max. 4.96	–	1.953 × 1.93 × 0.25 mm^3^	Silicon CMOS
^28^	Loop antenna	4/[65–69]	Max. 8	Max. 96.7	0.7 × 1.25 mm^2^	CMOS 0.18-μm
^29^	Dipole-antenna	7/[95–102]	Max. 4.8	–	–	Bi-CMOS
^30^	Tab monopole	30/[45–75]	Max. 0.1	Max. 42	1.5 × 1 mm^2^	Standard CMOS Silicon
^31^	Patch fed higher order mode DRA	25/[330–355]	Max. 7.9	Max. 74	0.2 × 0.5 mm^2^	0.18-μm SiGe
^32^	On-chip 3D (Yagi like concept)	40/[320–360]	Max. 10	Max. 80	0.7 × 0.7 × 0.43 mm^3^	0.13-μm SiGe
^33^	Half-mode cavity fed DRA	15/[125–140]	Max. 7.5	Max. 46	0.8 × 0.9 × 1.3 mm^3^	0.18-μm CMOS
^34^	Slot fed stacked DRA	10/[125–135]	Max. 4.7	Max. 43	0.9 × 0.8 × 1.5 mm^3^	0.18-μm CMOS
^35^	DRA	20/[120–140]	Max. 2.7	Max. 43	0.9 × 0.8 × 0.6 mm^3^	0.18-μm CMOS
^36^	8 × 8 magneto-electric dipole antenna array	14.7 [130.3–145]	Max. 20.5	Max. 59.2	32 × 20 × 0.818 mm^3^	LTCC
^37^	4 × 1 patch antenna array	32 [259–291]	Max. 5.2	–	2.47 × 1.53 × 0.675 mm^3^	0.675-μm GaN
^38^	2 × 1 octagonal shorted annular ring on-chip antenna array	17 [303–320]	Max. 4.1	Max. 38	0.55 × 0.5 × 0.3 mm^3^	0.13-μm SiGe BiCMOS
This work	Metasurface on-chip antenna	60/[410–470]	Min. 10	Min. 60	8.6 × 8.6 × 0.0503 mm^3^	Standard 50 μm GaAs layer

Freq. and Eff. represent frequency and efficiency, respectively.

## Conclusions

The study undertaken demonstrates the feasibility of an on-chip antenna constructed of an array of 11 × 11 circular patches at terahertz band. The antenna design is based on a metasurface which is fabricated on a thin but high-permittivity GaAs layer. The metasurface and the leaky-wave open-ended slot-lines feed structure are fabricated respectively on the top and bottom sides of the GaAs substrate layer. The proposed metasurface on-chip antenna is compact with dimensions of 8.6 × 8.6 × 0.0503 mm^3^ and it has an average gain that is in excess of 10 dBi and radiates with an average radiation efficiency in excess of 60% over frequency range of 0.41–0.47 THz.
